# Meteorological associations of *Vibrio vulnificus* clinical infections in tropical settings: Correlations with air pressure, wind speed, and temperature

**DOI:** 10.1371/journal.pntd.0011461

**Published:** 2023-07-06

**Authors:** Andrea J. Ayala, Ketty Kabengele, Salvador Almagro-Moreno, C. Brandon Ogbunugafor

**Affiliations:** 1 Department of Ecology and Evolutionary Biology, Yale University, New Haven, Connecticut, United States of America; 2 Burnett School of Biomedical Sciences, University of Central Florida, Orlando, Florida, United States of America; 3 National Center for Integrated Coastal Research, University of Central Florida, Orlando, Florida, United States of America; 4 Public Health Modeling Unit, Yale School of Public Health, New Haven, Connecticut, United States of America; 5 Santa Fe Institute, Santa Fe, New Mexico, United States of America; Johns Hopkins University Bloomberg School of Public Health, UNITED STATES

## Abstract

*V*. *vulnificus* is one of the deadliest waterborne pathogens, yet little is known of the ecological and environmental forces that drive outbreaks. As a nationally notifiable disease, all cases of *V*. *vulnificus* diagnosed in the United States are reported to the state in which they occurred, as well as to the Centers for Disease Control (CDC) in Atlanta, Georgia. Given that the state of Florida is a ‘hotspot’ for *V*. *vulnificus* in the United States, we examined the prevalence and incidence of cases reported to the Florida Department of Health (2008–2020). Using a dataset comprised of 448 cases of disease caused by *V*. *vulnificus* infection, we identified meteorological variables that were associated with clinical cases and deaths. Combined with data from the National Oceanic and Atmospheric Administration (NOAA), we first utilized correlation analysis to examine the linear relationships between satellite meteorological measurements such as wind speed, air temperature, water temperature, and sea-level pressure. We then measured the correlation of those meteorological variables with coastal cases of *V*. *vulnificus*, including the outcome, survival, or death. We also constructed a series of logistic regression models to analyze the relationship between temporal and meteorological variables during months that *V*. *vulnificus* cases were reported versus months when *V*. *vulnificus* cases were not reported. We report that between 2008 and 2020, *V*. *vulnificus* cases generally increased over time, peaking in 2017. As water temperature and air temperature increased, so too did the likelihood that infection with *V*. *vulnificus* would lead to patient death. We also found that as mean wind speed and sea-level pressure decreased, the probability that a *V*. *vulnificus* case would be reported increased. In summary, we discuss the potential factors that may contribute to the observed correlations and speculate that meteorological variables may increase in their public health relevance in light of rising global temperatures.

## Author summary

*V*. *vulnificus* is one of the deadliest waterborne pathogens in the world. It is a bacterium that is transmitted in aquatic settings and causes a serious human infection that can often end in death. Even though *V*. *vulnificus* has attracted recent attention from the public health sector, little is known about the environmental forces that contribute to outbreaks. In this study, we use data on *V*. *vulnificus* cases from the Florida Department of Health, and data from the National Oceanic and Atmospheric Administration (NOAA) to identify the meteorological factors that are associated with both cases and fatalities from *V*. *vulnificus*. We find that warmer air and water temperatures are associated with severe cases of *V*. *vulnificus*. In addition, our results suggest that decreasing wind speed and air pressure were associated with a higher probability of a *V*. *vulnificus* case. In the future, these kinds of data may help public health agencies mobilize resources in preventing outbreaks. Further, these results highlight the potential connection between the forces associated with climate change and infectious disease outbreaks.

## Introduction

Facultative pathogens are free-living microorganisms that are characterized by their ability to persist, reproduce, and transmit to susceptible hosts directly from their natural habitats [1]. Many are the causative agents of significant world-wide public health burdens, and yet, given their environmental sourcing, cannot be eradicated [2,3]. Some members of the Vibrionaceae, a family of aquatic bacteria, rank among the best known of facultative pathogens, with broad niche breadths and a nearly pan-coastal distribution [4]. Classified within the Vibrionaceae is *Vibrio vulnificus*, one of the most dangerous *Vibrio* species [5]. The bacterium is a halophilic, autochthonous inhabitant of aquatic environments that causes life-threatening septicemia in susceptible hosts, with a reported case fatality rate of up to 50 percent [5]. Although *V*. *vulnificus* is known for its worldwide distribution, epidemiological reports are most commonly linked to temperate coastal nations such as the United States, France, Germany, Israel, Korea, and Taiwan [6–8].

Living in Florida, and other states on the Gulf Coast (Texas, Louisiana, Mississippi, Alabama) is associated with an increased risk of contracting *V*. *vulnificus*, primarily between May and October [9–11]. *V*. *vulnificus* is also often isolated from locations where salinity ranges from 15 to 25 parts per thousand (ppt), and the water temperatures range from 9° to 31°C [10,12–14]. Prior studies performed in Florida have predominantly reported on the clinical features of infection, as opposed to analyzing the meteorological patterns that occur in association with case reports [5,15,16,17]. However, some studies have examined the relationship between *V*. *vulnificus* clinical cases and temperature [18,19]. Even before mandatory CDC reporting, cases were detected consistently between March and November, with a peak period of incidence in May [5,15]. Due to its high mortality rate, *V*. *vulnificus* was declared the most lethal foodborne illness in the state in 1993 [15]. Since then, the Florida Department of Health has maintained a reporting database, which has demonstrated an increased incidence of *V*. *vulnificus* clinical cases [20].

In addition to these studies of clinical infection, there are several examinations of the relationship between *V*. *vulnificus* abundance and temperature or seasonality. A study of four coastal habitats found that elevated sea surface temperatures and suspended particulate matter were variables that were predictive for detecting *V*. *vulnificus* both in water samples and oysters [21]. López-Pérez et al. (2021) reported recovering *V*. *vulnificus* in Florida more often during months when the water temperature exceeded 20°C [22]. With respect to seasonality, significant fluctuations were detected in the abundance of *V*. *vulnificus* in several aquatic organisms across several Gulf states [23,24]. Thus, we cannot overstate the importance of water and air temperature to the ecology of *V*. *vulnificus* [22,25]. Nonetheless, there remain many other abiotic factors which may contribute to *V*. *vulnificus* emergence that may contribute to poor clinical outcomes.

In this study, our objective was to establish associations between the incidence of clinical cases of *V*. *vulnificus* and standardized meteorological conditions: water temperature, windspeed, air temperature, and sea-level pressure. Using Florida as a model setting, we use a combination of case data from the Florida Department of Health in conjunction with data from the National Oceanic and Atmospheric Administration (NOAA) to identify several meaningful correlations. First, we examined correlations between our four variables of interest. We identified several significant associations between meteorological variables and the risk of infection. In closing, we speculate on the mechanisms underlying these associations and reflect on their relevance for conversations surrounding climate change and epidemiology.

## Materials and methods

### Terminology

We use the term “meteorological” to describe the factors that were used to diagnose correlations with clinical cases. The data come from satellite meteorological measurements and are key features of the microbial ecology that underlie *V*. *vulnificus* infections.

### Case acquisition and case fatality rates

Redacted data on *V*. *vulnificus* cases were kindly provided to the Ogbunu Lab at Yale University in December 2020 by the Florida Department of Health [16]. Pathogenic *Vibrio* illnesses became notifiable diseases to the Cholera and Other *Vibrio* Illness Surveillance (COVIS) system and Foodnet in late 2007. Since then, the state of Florida documented 448 cases of *V*. *vulnificus* between 1/1/2008 and 12/20/2020 that were acquired in the state and traceable to their originating county. Outcomes for each case, e.g., survival or death, were also provided in the dataset. To calculate the case fatality rate (CFR) from our 448 cases, we divided the total number of deaths, e.g., 111 that occurred between 2008 and 2020 by 448. We then multiplied the resulting ratio by 100 to yield a percentage.

### Clinical cases and meteorological data

**Fig 1:**
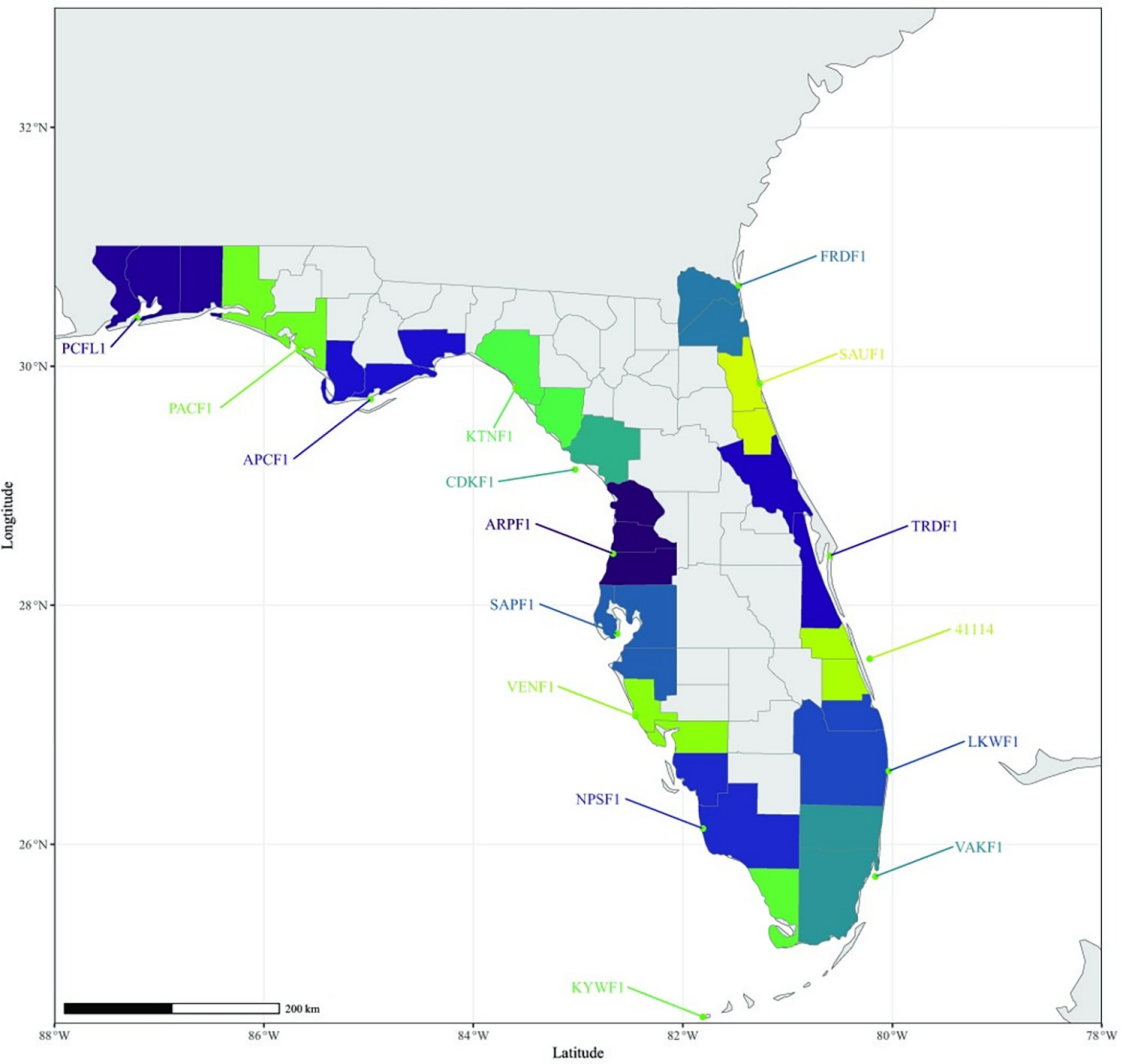
NOAA Coastal Buoys of Florida. Re-rendered from https://www.ndbc.noaa.gov/ on 12/22/2021. This demonstrates the distribution of the relevant NOAA coastal buoys (represented by green circles) around the coast of Florida, which collect meteorological data on coastal conditions every six minutes. Each buoy is represented by a five-letter code that correspond to counties filled in the same color. Corresponding county names are listed in Table 1.

In order to align meteorological information with our county case data, we utilized publicly available meteorological information from the global National Oceanic and Atmospheric Administration (NOAA) Marine Environmental Buoy Database and the NOAA National Buoy Data Center (NBDC) [26,27] (Fig 1). Florida’s NOAA buoys are distributed between two systems, the Gulf of Mexico-(West) and the Gulf of Mexico-(East), across a coastline length of 2,170 km. Each coastal buoy, regardless of system, collects standard meteorological data on a six minute interval basis for the following variables: year, month, date, hour, minute, wind, wind speed (WSPD) in meters/second, gust, wave height in meters, waves at the dominant period, average wave period in seconds, direction of the waves at the dominant period, sea-level pressure in hectopascals (PRES), air temperature in degrees Celsius (ATMP), water temperature in degrees Celsius (WTMP), dewpoint temperature in degrees Celsius, station visibility in nautical miles, pressure tendency and tide, e.g., the water level in feet above or below mean lower low water. Historical data is available for most NOAA buoys dating back to January 1^st^, 2005.

We corresponded NOAA buoys (Fig 1) to Florida coastal counties first by calculating which NOAA buoys were the closest to the middle of the coastline of the county, according to the centroid. Secondly, from this information, we identified which of the closest buoys maintained historical meteorological data dating back to 2008, the year that *V*. *vulnificus* cases were consistently reported to COVIS and FoodNet. Due to uneven coverage of the Florida coast by individual NOAA buoys, multiple coastal counties were often assigned a single buoy. Historical data (2008–2020) was extracted from NOAA, according to the corresponding buoy (Table 1), using the R programming language. Subsequently, the data were downloaded (as txt files) from each NOAA buoy’s page into the R statistical language platform for windspeed (WSPD), air temperature (ATMP), water temperature (WTMP) and sea-level pressure (PRES). Missing buoy data were indicated as 99 (WSPD), 999 (ATMP), 999 (WTMP) and 9999 (PRES) by NOAA. These values were removed from the imported data and means were calculated for all months (January to December) for each county, for the years of interest, 2008 to 2020.

**Table 1:** Names of NOAA buoys used in our analysis. The Florida counties that correspond to meteorological data, and NOAA reference information for each buoy.

NOAA Station (n = 16)	Coastal Counties (n = 34)	Reference
TRDF1/CapeCan	Brevard, Volusia	[28]
ARPF1/Aripeka	Pasco, Citrus, Hernando	[29]
PCLF1/Pensacola	Santa Rosa, Escambia, Okaloosa	[30]
41114/FortPierce	Indian River, St. Lucie	[31]
NPSF1/Naples	Lee, Collier	[32]
LKWF1/LakeWorth	Palm Beach, Martin	[33]
SAPF1/StPetersburg	Hillsborough, Pinellas, Manatee	[34]
FRDF1/Fernandina	Duval, Nassau	[35]
VAKF1/VirginiaKey	Broward, Dade	[36]
SAUF1/StAugustine	St. Johns, Flagler	[37]
VENF1/Venice	Sarasota, Charlotte	[38]
PACF1/Panama	Bay, Walton	[39]
APCF1/Apalachicola	Franklin, Wakulla, Gulf	[40]
KYWF1/KeyWest	Monroe	[41]
KTNF1/Keaton	Taylor, Dixie	[42]
CDRF1/CedarKey	Levy	[43]

Data were only analyzed from each NOAA buoy for the year that a case occurred in its corresponding county. For months when no cases were reported, we also collected the monthly mean of the listed variables. This provided the opportunity to compare the meteorological conditions that occurred during months when cases were reported, and during months when cases were not reported.

### Inclusion criteria for meteorological variables

Note that out of all the data collected by the NOAA buoys, we only extracted and analyzed correlations with wind speed (WSPD), air temperature (ATMP), water temperature (WTMP), and sea-level pressure (PRES). As first proposed in the introduction, these are related to factors associated with *Vibrio* populations in other contexts. Rather than analyze correlations between all available NOAA meteorological variables, our approach was more directed and hypothesis-driven, utilizing only those meteorological measurements that might have a relationship with *Vibrio* populations [22]. Below we describe the variables and their associations.

- *WSPD*, *PRES (storm events)*. Tropical cyclones have been linked to a rise in *V*. *vulnificus* cases [44–47]. After Hurricane Katrina made landfall in Louisiana, extensive flooding in the city of New Orleans was linked to a spike in *V*. *vulnificus* clinical cases [44]. A longitudinal study of Lake Pontchartrain post-Hurricanes Katrina and Rita in New Orleans found that *V*. *vulnificus* abundance increased closer to the lake shoreline, and was positively correlated to increasing temperature, turbidity, and salinity [46]. Interestingly, sampling in the Chesapeake Bay area of Maryland, USA, found no significant change in the concentration of *V*. *vulnificus* in samples collected from the top of the water column and in oysters before and after Hurricane Irene impacted the area, likely as a result of the ensuing wave action and sediment resuspension [48,49]. In North Carolina, data on the abundance of *V*. *vulnificus* within the Neuse River Estuary was collected before and after two major storm events–Hurricane Ophelia and Tropical Storm Ernesto [45]. Unexpectedly, the prevalence of *V*. *vulnificus* in storm samples varied, from Tropical Storm Ernesto, with an 80% prevalence in storm samples, to Hurricane Ophelia, with a 31% storm sample prevalence [45].
- *WTMP*, *and ATMP (elevated temperature)*. Water temperature has an established relationship with *V*. *vulnificus* density and abundance. Elevated water temperature has the highest association with *V*. *vulnificus* abundance, while other variables, such as increased turbidity, low dissolved oxygen concentrations, increased estuarine bacterial levels and high fecal coliform levels are also predictive for the detection of *V*. *vulnificus* [50]. Though most of the literature has focused on water temperature, we wanted to test if there was a relationship with air temperature.
- *Seasonality* (the number of cases per season). In the northeastern United States, Tilton and Ryan (1987) sampled water and shellfish, specifically mussels, oysters, clams, and whelk, for the presence and abundance of *V*. *vulnificus* during the spring and summer months [19]. No *V*. *vulnificus* could be isolated from water samples and shellfish until the temperature reached 17°C, plateauing when temperatures reached 22°C [19].

### Statistical analyses

All statistical analyses were performed using the R statistical language (version 4.1.2) [41]. We assessed the normality of our data distributions for Seasonality, WSPD, PRES, ATMP, and WTMP using the Shapiro-Wilks test of Normality, with a p-value threshold of 0.05 [42]. To determine the strength of the relationship between each meteorological variable, we performed a series of non-parametric Spearman’s Rank Correlation ρ coefficients to measure their association. As our meteorological variables are in a continuous data format, Spearman’s Rank Correlation ρ is the most appropriate test to measure associations between the data. We performed the analyses covering four conditions: all periods, e.g., during months when no cases were reported, during months when cases were reported, and during months when deaths were reported as one dataset. Then, we subsetted the data for ‘all periods’ to examine the strength of those relationships during each condition: months when no cases were reported, months when cases were reported, and months when deaths were reported. Subsequent analyses of stratified data were performed using the non-parametric Kruskal-Wallis H test for three independent groups [43], and the Mann-Whitney U test for two independent groups [44], both with a p-value threshold of 0.05 for statistical significance.

After performing our Shapiro-Wilks test of Normality, the associations between our meteorological variables and seasonality were assessed using the non-parametric Chi-Square Goodness of Fit test. Although our meteorological variables are in a continuous data format, seasonality is represented by categorical data, e.g., winter, spring, summer, and fall. This statistical test allowed us to examine whether a given phenomenon, i.e., seasonality of the reported cases, adhered to a proposed distribution of the data. Specifically, we asked whether each season deviated from a distribution where cases were distributed across seasons equally.

To determine the model that best described the system, we ran a series of logistic regressions using combinations of our meteorological variables coupled with NOAA reporting stations, and their corresponding counties, as well as Month, and Season as our variables of interest. Logistic regression was utilized by coding months when cases occurred as one, and months when cases did not occur as zero [28]. We used AIC and McFadden’s pseudo R^2^ to identify the model that accounts for the predominance of the variation seen in our data [45,46].

## Results

### Descriptive analysis of coastal and non-coastal cases combined: Seasonality, county occurrence, annual reporting, and monthly analysis

**Fig 2:**
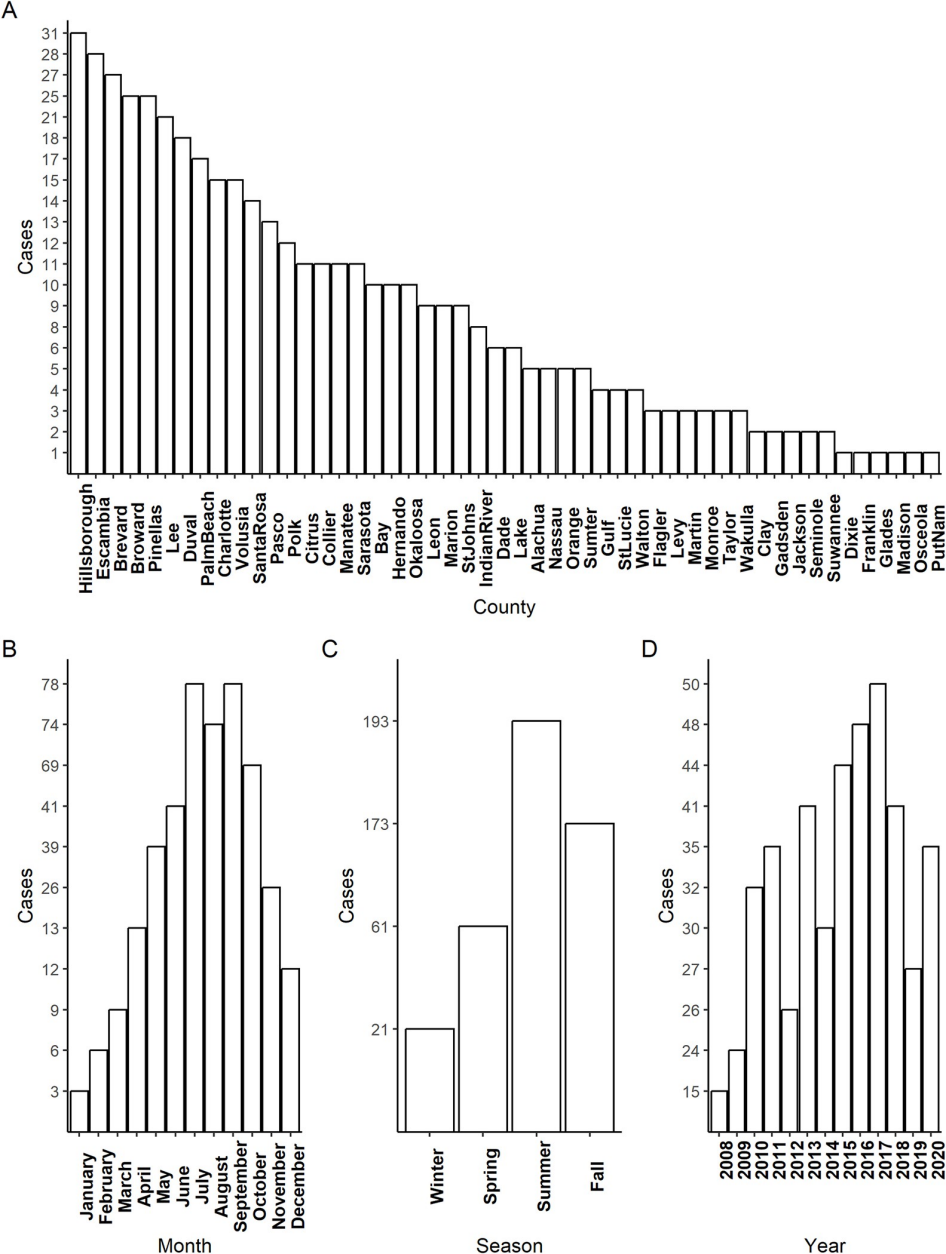
Occurrences of *V*. *vulnificus* in Florida between 2008 and 2020. Panel A depicts the relative occurrences of the 448 cases across each of Florida’s 50 reporting counties documented between 2008 and 2020. The data is in descending order of frequency by county, with the county of Hillsborough demonstrating the highest reported occurrences of *V*. *vulnificus* in the state. Panel B demonstrates the breakdown of V. vulnificuscases in Florida by month, between the years 2008 and 2020 in the state of Florida. July, August, and September demonstrate the highest incidence of reporting. Panel C breaks down our dataset of 448 cases according to season. Summer (June, July, and August) had the greatest number of reported cases, followed by fall (September, October, and November), with the fewest cases reported in the winter (December, January, and February), followed by spring (March, April, and May). Panel D represents our dataset of 448 cases broken down according to year. In 2008, the fewest cases were reported, while in 2017, the highest frequency of cases was reported.

A total of 456 cases were reported between 1/1/2008 and 12/20/2020 to the Florida Department of Health. Eight cases were unconfirmed with respect to having been acquired in Florida, and thus were removed from the dataset, leaving 448 cases. *V*. *vulnificus* cases were reported in 50 of 67 counties in the state of Florida during the study period (Fig 2A). Counties reporting equal to or greater than 25 cases over the twelve-year period were: Hillsborough, Escambia, Brevard, Broward, and Pinellas. Counties reporting the fewest cases (one case each) were: Dixie, Franklin, Gadsden, Madison, Osceola, and Putnam.

We also found that cases were reported most frequently during the months of July and September (Fig 2B) during the period spanning from 2008 and 2020, with the fewest cases occurring in January and February. Seasonality as a variable was also assessed (Fig 2C), with the most cases reported in the summer months (June, July, and August) and the fewest in the winter (December, January, and February). The Shapiro-Wilk’s Test of Normality demonstrated that the data for seasonality was normally distributed (W = 0.88475, p-value = 0.3593). Nonetheless, we utilized a non-parametric Chi-Square Goodness of fit test, given our question, “Are cases distributed across seasons unequally?” Our sample sizes for each season were: Winter, n = 21, Spring, n = 61, Summer, n = 193, and Fall, n = 173. This led to a significant Chi-Square χ^2^ = 3, df = 3, p-value = <0.0001, indicating that seasonality was statistically significant. We followed up this omnibus Chi-Square Goodness of Fit test with sequential Chi-Square Goodness of Fit Tests, corrected using a Bonferroni correction adjusted to a p-value of 0.00833. Winter-spring was significantly reported as χ^2^ = 19.512, df = 1, p-value = <0.0001, winter-summer was significantly reported as χ^2^ = 119.09, df = 1, p-value = <0.0001, winter-fall was significantly reported as χ^2^ = 138.24, df = 1, p-value = <0.0001, spring-summer was significantly reported as χ^2^ = 54.607, df = 1, p-value = <0.0001, spring-fall was significantly reported as χ^2^ = 68.598, df = 1, p-value = <0.0001, and summer-fall was not significant at χ^2^ = 1.0929, df = 1, p-value = 0.2958.

When trends were separated by year (Fig 2D), they demonstrate that the fewest cases were reported in 2008, and the largest number in 2017. In general, cases increased on an annual basis. Specifically, in 2008, n = 15 cases were reported, and in 2017, n = 50 cases were reported, for an increase of 233% (S1 Fig) between the lowest and highest reporting yearly incidence in Florida.

### Overall case fatality rate and mortality and case fatality rate by month, season, and year

We calculated the overall case fatality rate (CFR) from our 448 cases, of which 111 cases had death as an outcome. This yielded a CFR of 24.78% across the state of Florida in our given time period [47]. When categorized by month, we found that like our case occurrence data, the overall deadliest months were September (21 deaths), followed by July (19 deaths). The fewest deaths occurred in February (0 deaths), which were followed by January, March, and December (2 deaths). CFRs by month are given in Table 2.

**Table 2:** Case fatality rates of *V*. *vulnificus* as reported by the state of Florida. In general, case fatality rates did not exceed 30 percent, except for January and April.

Month	Case Fatality Rate	Number of Cases	Number of Deaths
January	66.67%	3	2
February	0	6	0
March	22.22%	9	2
April	30.77%	13	4
May	28.21%	39	11
June	24.39%	41	10
July	24.36%	78	19
August	24.32%	74	18
September	26.92%	78	15
October	21.74%	69	15
November	26.92%	26	7
December	16.67%	12	2

Our computed CFRs suggested that cases were more likely to have a negative outcome from infection during the months of January, followed by April. However, note that that the CFR data for winter months have limited sample sizes. Therefore, we need to be careful in our interpretations. When categorized by season, the data showed that the case fatality rate of the fall was 24.86%, the CFR of the spring was 27.87%, the CFR of the summer was 24.35%, and the CFR of the winter was 19.05%. Yearly CFRs were as follows: 2008 (40%), 2009 (29.17%), 2010 (31.25%), 2011 (34.28%), 2012 (34.62%), 2013 (29.27%), 2014 (16.67%), 2015 (29.55%), 2016 (20.83%), 2017 (22%), 2018 (17.07%), 2019 (7.4%), and 2020 (20%).

### Non-parametric Spearman’s Rank Correlation tests

We found that many variables had statistically significant relationships according to a p-value of 0.05 across all stratifications, i.e., all data points, then subsetted across cases, no cases, and deaths (Table 3). However, the strengths of those correlations were weak except for ATMP and WTMP, where a strong relationship was defined as one with a ρ of greater than 0.80. Notably, WSPD was statistically significant and had a higher ρ correlation value with ATMP and WTMP during months when cases and deaths were reported, as opposed to when no cases were reported. Given the negative values attributed to the ρ values overall, this suggests that as WSPD increased, WTMP and ATMP decreased during months when cases and deaths were reported. In addition, WSPD and PRES were statistically significant during months when no cases were reported.

**Table 3:** Spearman’s correlation coefficients for our meteorological variables. To determine the strength of the relationship between each meteorological variable, we performed a series of non-parametric Spearman’s Rank Correlation ρ coefficients to measure their association. We performed the analyses covering four conditions: all periods, e.g., during months when no cases were reported, during months when cases were reported, and during months when deaths were reported as one dataset. While many of the variables had statistically significant relationships according to the p-value, those correlations’ strengths were weak, except for ATMP and WTMP, where a strong relationship was defined as one with a ρ of greater than 0.80.

Condition	Variable 1	Variable 2	S	p-value	Spearman’s Rank Correlation ρ	df
All	WSPD	ATMP	1075558076	0.0004096	-0.08290868	1811
All	WSPD	WTMP	1047396443	0.02018	-0.05455459	1811
All	WSPD	PRES	1047395100	0.02018	-0.05455323	1811
All	ATMP	WTMP	41404884	<0.0001	0.9583121	1811
All	ATMP	PRES	1635063628	<0.0001	-0.646238	1811
All	WTMP	PRES	1637838280	<0.0001	-0.6490316	1811
No cases	WSPD	ATMP	645444236	0.1158	-0.03995624	1548
No cases	WSPD	WTMP	6.26e+08	0.7344	-0.008625618	1548
No cases	WSPD	PRES	662269930	0.00826	-0.06706622	1548
No cases	ATMP	WTMP	25200928	<0.0001	0.9593956	1548
No cases	ATMP	PRES	1029502253	<0.0001	-0.6587603	1548
No cases	WTMP	PRES	1030097863	<0.0001	-0.65972	1548
Cases	WSPD	ATMP	3778459	<0.0001	-0.2462496	261
Cases	WSPD	WTMP	3701511	0.0003068	-0.2208699	261
Cases	WSPD	PRES	3222070	0.3108	-0.06273555	261
Cases	ATMP	WTMP	325697	<0.0001	0.8925753	261
Cases	ATMP	PRES	4064933	<0.0001	-0.3407373	261
Cases	WTMP	PRES	4092443	<0.0001	-0.3498109	261
Deaths	WSPD	ATMP	34962	0.05402	-0.2612656	53
Deaths	WSPD	WTMP	35597	0.03549	-0.2841782	53
Deaths	WSPD	PRES	27074	0.866	0.02328938	53
Deaths	ATMP	WTMP	4681.5	<0.0001	0.8311145	53
Deaths	ATMP	PRES	35431	0.03973	-0.2781897	53
Deaths	WTMP	PRES	32235	0.2348	-0.1628633	53

### Meteorological data in correlation with case and mortality reporting

Given that NOAA buoys are only located along coastal regions, we analyzed how data from the buoys corresponded to cases that originated only in coastal counties. This left a total of 383 cases for analysis, representing 34 counties whose meteorological variables were measured by 16 buoys (Table 1). Each individual county potentially represented 12 months of data per year. When accounting for multiple counties and missing data, there were 1,813 total months during years when cases were reported for which we had meteorological data. A total of 263 of those months were months when cases were reported (n = 263). However, when accounting for multiple counties and missing data, there were 1,550 total months during years when cases were not reported. We then stratified WSPD (wind speed), ATMP (air temperature), WTMP (water temperature), and PRES (sea-level pressure) across all coastal counties according to the months in a case year when no cases were reported, during months when cases with a survival outcome were reported, and during months when cases with a fatal outcome were reported for the following analyses.

#### (a) Windspeed (WSPD)

**Fig 3:**
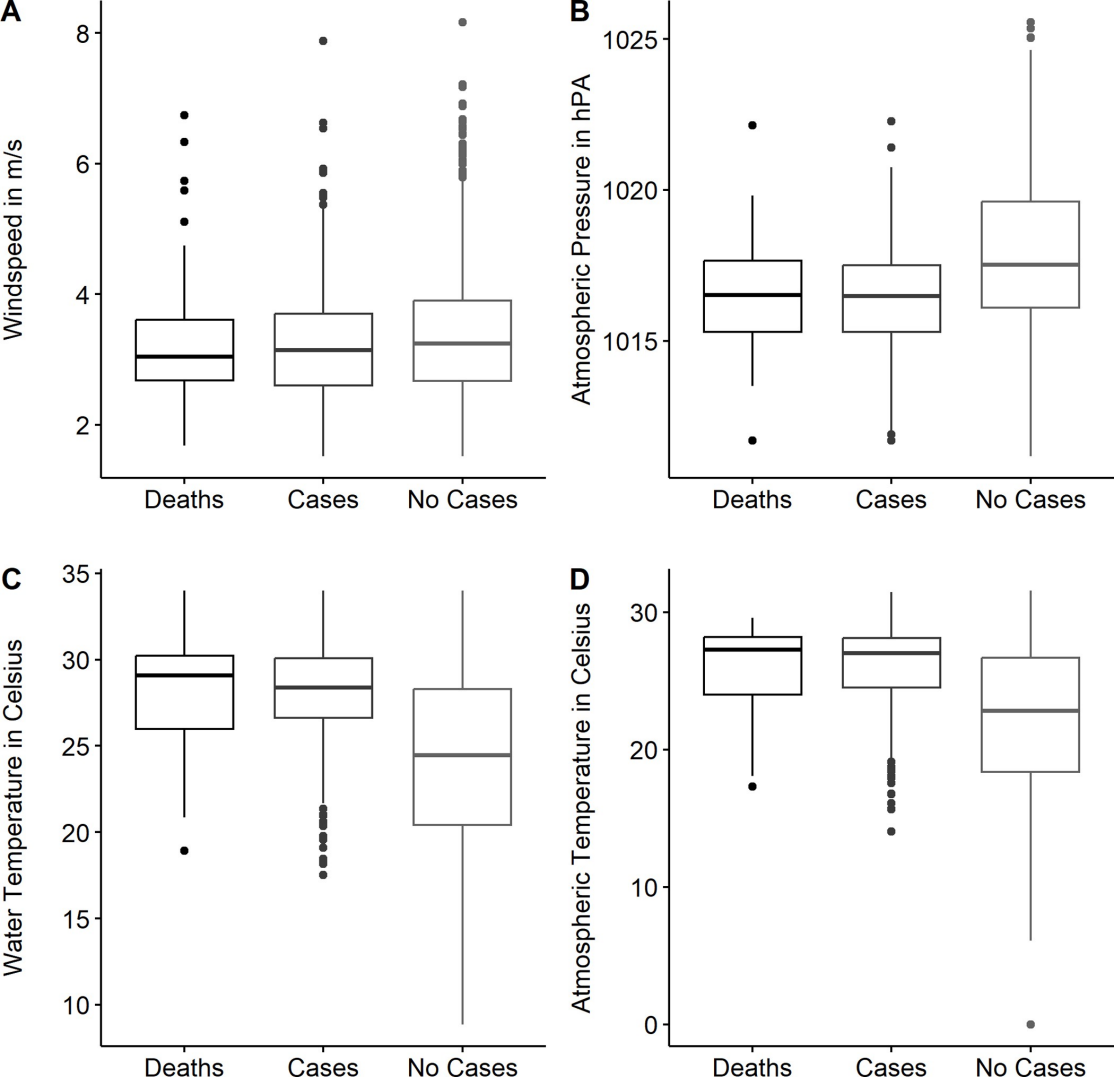
Correlations between various environmental variables and cases of *Vibrio vulnificus*. A) windspeed (WSPD) in meters/second versus the incidence of *V*. *vulnificus* cases reported in Florida, stratified by *V*. *vulnificus* incidence, specifically during months when no cases were reported, months when cases with a survival outcome were reported, and months when mortality due to *V*. *vulnificus* infection was reported. The mean WSPD for months when no cases were reported was 3.35 ± 0.02 SE m/s, while the mean WSPD for months when cases where survival was the outcome was a mean of 3.26 ± 0.05 SE m/s, and the mean WSPD for months when death was the outcome was 3.27 ± 0.10 SE m/s. The Mann-Whitney U test indicated that the differences between the two groups were statistically significant (W = 325379, p-value = 0.01527). B) sea-level pressure (PRES) is stratified by *V*. *vulnificus* incidence according to months reporting deaths, months reporting cases, and months reporting no cases. The mean PRES for months when no cases were reported was 1017.8 ± 0.005 SE hPa while the mean PRES for months when cases where survival was the outcome was a mean of 1016.5 ± 0.003 SE hPa, and the mean PRES for months when death was the outcome was 1016.5 ± 0.002 SE hPa. C) Water temperature stratified by the reporting of *V*. *vulnificus*, specifically during months when no cases were reported, months when cases with a survival outcome were reported, and months when mortality due to *V*. *vulnificus* was reported. We found that our WTMP Kruskal-Wallis test was significant for this analysis, (χ2 = 176.14, df = 2, p-value < 0.0001), however, similar to ATMP, a pairwise multiple comparisons test with a Bonferroni adjustment demonstrated no significant difference between WTMP when survival cases were reported, and WTMP when non-survival cases were reported (p-value = 0.25). D) Air temperature (ATMP) stratified by *V*. *vulnificus* incidence, specifically during months when no cases were reported, months when cases with a survival outcome were reported, and months when a mortality due to *V*. *vulnificus* was reported. As ATMP increases, so too does the likelihood of the severity of *V*. *vulnificus* cases. While this was a significant relationship (ATMP Kruskal-Wallis χ2 = 181.32, df = 2, p-value < 0.0001), a follow-up pairwise multiple comparison test with a Bonferroni adjustment demonstrated that the differences in ATMP between months when cases were reported and when cases were not reported was significant (p<0.0001), but not during months when survival cases were reported, and non-survival cases were reported (p = 0.72). Please see main text for additional details.

The mean WSPD for months when no cases were reported was 3.35 ± 0.02 SE m/s (median = 3.24 m/s), while the mean WSPD for months when cases where survival was the outcome was a mean of 3.26 ± 0.05 SE m/s (median = 3.12 m/s), and the mean WSPD for months when death was the outcome was 3.27 ± 0.10 SE m/s (median = 3.04 m/s). Using the Shapiro-Wilks test of Normality, we found that WSPD deviated from a Gaussian distribution for all stratifications (W = 0.96096, p-value < 0.0001) when no cases were reported, (W = 0.89122, p-value = <0.0001) when survival was reported, and (W = 0.89771, p-value = <0.0001) when death was reported. Given that there was little difference between the means of months of *V*. *vulnificus* cases when deaths or survival were reported, we pooled our case data to perform a non-parametric Mann-Whitney U test to compare the means between the WSPD of the months when cases were reported, and months when cases were not reported. The Mann-Whitney U test indicated that the differences between the two groups were statistically significant (W = 325379, p-value = 0.01527), with the mean WSPD increased during the months that no cases were reported in contrast to months when cases were reported, regardless of their outcome (Fig 3A). This suggests that as WSPD increases, the number of cases reported decreases.

#### (b) Air Temperature (ATMP)

The mean ATMP for months when no cases were reported was 22.1 ± 1.15 SE °C, while the mean ATMP for months when cases where survival was the outcome was a mean of 25.9 ± 0.17 SE °C, and the mean ATMP for months when death was the outcome was 27.9 ± 0.36 SE °C (Fig 3D). Using the Shapiro-Wilks test of Normality, we found that ATMP deviated from a Gaussian distribution for all stratifications (W = 0.94786, p-value < 0.0001) when no cases were reported, (W = 0.86938, p-value = <0.0001) when survival was reported, and (W = 0.83798, p-value = <0.0001) when death was reported.

Thus, given that mean ATMP was highest for death, followed by survival, and lastly for months when no cases were reported, we assessed the differences between the three groups using a non-parametric Kruskal-Wallis test of independent groups. While this was significant (Kruskal-Wallis χ^2^ = 181.32, df = 2, p-value < 0.0001), a follow-up pairwise multiple comparison test with a Bonferroni adjustment demonstrated that the differences in ATMP between ATMP during months when cases were reported and cases were not reported was significant (p<0.0001), but not during months when survival cases were reported, and non-survival cases were reported (p = 0.72). Since this test was not significant, we pooled our case data to test the mean of the two independent groups, utilizing the Mann-Whitney U test to compare the means between the ATMP of the months when cases were reported, and months when cases were not reported. The Mann-Whitney U test indicated that the differences between the two groups were statistically significant (W = 508627, p-value = <0.0001), with the mean ATMP decreased during the months that no cases were reported, in contrast to months when cases were reported, regardless of their outcome. Overall, there appears to be a strong correlation between increased air temperature and *V*. *vulnificus* infections that lead to patient death.

#### (c) Water Temperature (WTMP)

The mean WTMP for months when no cases were reported was 24.5 ± 0.02 SE °C, while the mean WTMP for months when cases where survival was the outcome was a mean of 27.9 ± 0.17 SE °C, and the mean WTMP for months when death was the outcome was 28.2 ± 0.33 SE °C (Fig 3C). To assess whether the distribution of each stratification deviated from the Gaussian distribution, we utilized the Shapiro-Wilks test of Normality. The data showed that WTMP had a non-normal distribution for all stratifications, e.g., (W = 0.96311, p-value < 0.0001) when no cases were reported, (W = 0.91159, p-value = <0.0001) when survival was reported, and (W = 0.92052, p-value = <0.0001) when death was reported.

Given that mean WTMP was highest for death, followed by survival, and lastly for months when no cases were reported, we assessed the differences between the three groups using a non-parametric Kruskal-Wallis test of independent groups. We found that our Kruskal-Wallis test was significant for this analysis, (Kruskal-Wallis χ^2^ = 176.14, df = 2, p-value < 0.0001), however, similar to ATMP, a pairwise multiple comparisons test with a Bonferroni adjustment demonstrated no significant difference between WTMP when survival cases were reported, and WTMP when non-survival cases were reported (p-value = 0.25). We followed up this analysis by pooling our observations for WTMP when cases were reported, regardless of the outcome, in contrast to months when no cases were reported. A Mann-Whitney U test was performed, and this test was significant between the two independent groups (W = 451022, p-value < 0.0001). Overall, the data indicates that there appears to be a strong correlation between increased air temperature and *V*. *vulnificus* infections that lead to patient death.

#### (d) Pressure at Sea level (PRES)

The mean PRES for months, when no cases were reported, was 1017.8 ± 0.005 SE hPa while the mean PRES for months when cases, where survival was the outcome, was a mean of 1016.5 ± 0.003 SE hPa, and the mean PRES for months when death was the outcome was 1016.5 ± 0.002 SE hPa (Fig 3B). We examined whether each stratification’s distribution deviated from the Gaussian distribution using the Shapiro-Wilks test of Normality. The data showed that PRES had a non-normal distribution for one of three stratifications, e.g., (W = 0.988, p-value < 0.0001) when no cases were reported, (W = 0.98942, p-value = 0.01721) when survival was reported, and (W = 0.98437, p-value = 0.4756) when death was reported. Given that mean PRES was extremely similar for months when cases survived, and when cases did not survive, we pooled our observations for PRES when cases were reported, regardless of the outcome, in contrast to months when no cases were reported. A Mann-Whitney U test was performed, and this test was significant between the two independent groups (W = 227799, p-value < 0.0001). In general, this suggests that as PRES decreases, the likelihood of *V*. *vulnificus* case reporting decreases.

### Combining the meteorological factors into single models: Logistic regression of variables

We observed significant correlations between two of our meteorological variables and denoted that all four variables had significant associations with *V*. *vulnificus* case reporting. Thus, we hypothesized that a statistical model composed of a combination of these variables would account for the variation in the reporting of clinical cases across the study sample. To do this, we used a logistic regression approach to compare different models.

Our logistic regression utilized AIC and McFadden’s R^2^ to identify the most parsimonious model that accounted for the variation in the likelihood of case occurrence seen in our data. The data suggested that the best model only accounted for 14.1% of the total variance. This model was generated by the independent variables WSPD + ATMP + WTMP + PRES + Month, with an AIC value of 1322.3 (Table 4). Month was a categorical variable with 12 levels, thus the months that generated a significant p-value were July (p-value = 0.015436), August (p-value = 0.013719), September (p-value = 0.0004), October (p-value = <0.0001), and November (p-value = 0.031814). In effect, although individual meteorological factors were correlated with clinical cases, combinations of these factors into a single model only explained, at best, roughly 14% of the variation in case reporting.

**Table 4:** Listed in this table are the models used to assess the meteorological, spatial, and temporal variables in a series of logistic regressions. Also listed are their corresponding AIC values, McFadden’s R^2^, model degrees of freedom, and null deviance. *Corresponds to the best-identified model.

Logistic Model	AIC	McFadden’s R^2^	Model df	Null Deviance
WSPD + ATMP + WTMP + PRES	1346.1	0.1100731 (df = 5)	1812	1501.3
WSPD + ATMP + WTMP + PRES + County	1370.7	0.1309515 (df = 33)	1812	1501.3
WSPD + ATMP + WTMP + PRES + NOAA Station	1354.9	0.1201839 (df = 17)	1812	1501.3
WSPD + ATMP + WTMP + PRES + Month	1322.2	0.140644 (df = 16)	1812	1501.3
WSPD + ATMP + WTMP + PRES + Season	1322.8	0.1295665 (df = 8)	1812	1501.3
ATMP + WTMP + PRES + Season	1320.9	0.1295378 (df = 7)	1812	1501.3

## Discussion

In 1999, Linkous and Oliver stated, “While *V*. *vulnificus*alone is responsible for 95% of all seafood-related deaths in the United States, it is still a mystery why more people do not develop this infection [11].” Balasubramanian et al. (2022) reported that biotic and abiotic factors generate selective pressures in ecosystems, which facilitate the emergence of pathogenic traits in *V*. *vulnificus* [51]. Thus, in the present study, we assessed the association between seasonality and meteorological variables such as wind speed (WSPD), air temperature (ATMP), water temperature (WTMP), and pressure at sea level (PRES) in comparison to the reporting of *V*. *vulnificus* cases. It was not surprising that WTMP and ATMP were both significantly correlated with one another and demonstrated a strong relationship according to their ρ correlation value, regardless of whether cases were reported in a month. More surprising patterns involving WSPD: correlations were statistically significant with a higher ρ correlation value with ATMP and WTMP during months when cases and deaths were reported (as opposed to when no cases were reported). Given the negative values attributed to the ρ values, this suggests that as WSPD increased, WTMP and ATMP decreased during months when cases and deaths were reported. It was also interesting to note that WSPD and PRES were statistically significant during months when no cases were reported.

In addition, each of these was statistically significant when analyzed univariately, but none more than WTMP. On the other hand, PRES and WSPD were negatively correlated with *V*. *vulnificus* cases. These did not, however, account for explaining a notable amount of variation in the larger logistic regression model. Given that our meteorological variables, coupled with the variable ‘Month’ account for only 14.1% of the variance of the likelihood between a case being reported versus not being reported, approximately 85% of the variance remains to be explained. This suggests these three variables are not sole predictors of *V*. *vulnificus* cases, and that the true cause of disease emergence likely contains many more drivers. This is consistent with modern understandings of disease emergence as being a complex system, composed of interactions between molecular and ecological factors [51–54]. We speculate that epidemiological and microbial factors, such as host susceptibility and strain of *V*. *vulnificus*, may be more important to the development of an infection as opposed to the meteorological variables that we measured.

The meteorological conditions that we assessed, especially WTMP and ATMP, may not only have influenced the distribution and growth of *V*. *vulnificus* but also the behavior of human hosts in Florida. For example, warmer temperatures may promote the presence and growth of *V*. *vulnificus* in a body of water, but these conditions, coupled with the season, may also encourage individuals to consume raw seafood or encounter coastal waters. Other environmental variables not measured in this analysis, such as pH and salinity, may contribute to the explanatory variance in a logistic model [55,56]. Indeed, these highly significant variables, including rainfall events, have been reviewed in-depth in the literature as they relate to *V*. *vulnificus* and other *Vibrio* species [22,57,58]. In addition, we note that the assignment of our 34 coastal counties to only 16 buoys may have reduced our variance.

In our dataset, peak infections were reported in the late summer and fall, likely due to the water temperature around coastal Florida at that time. In our preliminary analysis of all 448 cases, *V*. *vulnificus* cases were most frequently reported in the months of July through September. When correcting for coastal cases that contained corresponding meteorological variables, we found that mean WTMP was highest during those months when *V*. *vulnificus* was most frequently reported, e.g., July (30°C), August (30.1°C), and September (29.3°C). This data dovetails with the literature. In 1998, Motes et al. calculated the density of *V*. *vulnificus* in Atlantic coast oysters (*Crassostrea virginica*) from sites located on the Gulf Coast in contrast to the Atlantic. *V*. *vulnificus* in oysters from the Gulf Coast peaked between the months of May and October, which is similar to the frequency of human cases of *V*. *vulnificus* reported across the coastal cases of Florida in our dataset [14]. In areas of the globe where *V*. *vulnificus* has been detected or reported in water or shellfish, a correlation between water temperature and the density and/or abundance of *V*. *vulnificus* has been repeatedly demonstrated [19,20,24,25,50,59–64]. In our present study, we also show an association between water temperature and the incidence of *V*. *vulnificus* clinical cases. This, however, begets the question, how will the incidence of *V*. *vulnificus* change over time as global climate change causes sea surface temperatures to continually increase [65]?

In Israel, Paz et al. (2007) examined the association between climate change, specifically warming temperatures, and the increased incidence of *V*. *vulnificus* in fish workers [59]. Since then, the topic of climate change and its relationship to *V*. *vulnificus* has garnered increasing attention [66–69]. Deeb et al. reported that the incidence of all *Vibrio* infections has risen by 41% in the decade before 2005 [65]. In our dataset, we report an increase of 233% between 2008 and 2017. Climate change is a crucial avenue of research, given that sea surface temperatures, tropical cyclone activity, and precipitation are all expected to increase in the Gulf of Mexico by the year 2100 [70].

While it is difficult to directly measure the impact of hurricanes on the density of a virulent species of bacteria using our monthly case report approach, we were able to indirectly measure the effect of extreme storm events using the measurements of sea-level pressure (PRES), which decreases during storm events [71,72]. In our univariate analyses, we found that PRES was negatively correlated with the frequency of *V*. *vulnificus* cases,–however, its effect was dampened when used to build larger logistical models. As the number of hurricane days during the summer and fall months increases in the state of Florida [48,73,74], we predict that this trend will be more apparent.

Even when indirectly measured, a trending correlation between climate change factors and the frequency with which *V*. *vulnificus* cases are reported in Florida is evident. For instance, Klontz et al. (1988) reported 62 cases in Florida between 1981 and 1987, for a mean of 8.8 cases per year [5]. In this study, 37.3 cases were reported on average per year, for a total of 448 cases between 2008 and 2020. That number jumped to 50 cases in 2017, the year that the highest frequency of cases was reported, with 22% of those cases reporting death as an outcome (11 deaths). For comparison, in 2013, the CDC reported that between 2002 and 2007, per year, 95 cases were reported with 85 hospitalizations and 35 deaths globally [12].

Within Florida, our data demonstrate that there are counties that disproportionately account for most of the clinical cases that were reported. The top five reporting counties were Hillsborough, Escambia, Pinellas, Brevard, and Broward. The former three lie on the Gulf coast of Florida, Brevard lies on the Atlantic coast, and Broward in southwestern Florida. While we can report on the patterns, our dataset cannot account for these spatial differences. More research into the current dataset is needed to determine whether the strains that lie off the coast of these counties have the potential to be more virulent in human hosts, than in counties where fewer cases have been reported. Interestingly, a similar trend was detailed by López-Pérez et al. 2021, who reported differences in the virulence of *V*. *vulnificus* populations along the Atlantic coast of Florida [22]. Lastly, another reason for the rise in case reporting may be due to increased detectability via medical workers [7].

Finally, our case fatality rates were much lower than those reported in the literature [9,75,76], for a mean of 24.78% across our 448-case dataset. Bross et al. reported case fatality rates of 50 percent for primary septicemia, and 15 percent for wound infections [77]. Klontz et al. noted a slightly higher trend for earlier wound infections in Florida, for a case fatality rate of 24% [5]. Given that our dataset did not distinguish between primary septicemia and wound infections, it is difficult to resolve why our case fatality rates were relatively low. One reason may be that medical personnel in Florida are now more attuned to the clinical signs of the disease than when it was initially characterized. Another may be that wound septicemia is the primary presentation, as opposed to the consumption of contaminated oysters, which carries a higher risk of mortality [15]. This supposed deviation should not surprise us, however, as *V*. *vulnificus* has already been demonstrated to have notable demographic and comorbidity factors that influence the development of disease [1,2,77]. It is also important to note that our reported case fatality ratios are limited to further extrapolation based on sample size, especially in the months of January and April. And while our study identifies several key environmental actors that play a role in disease, further work is needed for truly holistic, multidimensional predictive models of *V*. *vulnificus* transmission and death.

## Conclusion

In this study, we have orchestrated a data science approach that combines reliable data from two different government sources—public health (Florida Department of Health) and meteorological (NOAA)—to establish correlations between environmental factors and clinical cases of disease caused by *V*. *vulnificus*. The findings reinforce the importance of the environmental dimension of disease emergence, where the infection process is just the final step in a multi-step process with multiple actors and forces. In particular, the relationship between variables associated with elevated temperature and storm conditions is germane to conversations about how climate change may influence disease emergence and implore the development of more rigorous models that incorporate multiple actors in our effort to predict outbreaks of emerging diseases like *Vibrio vulnificus*. More generally, our findings further support the notion that disease emergence should be considered a complex system and understood with respect to interactions between a multitude of actors.

## Supporting information

S1 Fig *V*. *vulnificus* cases and percentage change over time. Here we demonstrate that the fewest *V*. *vulnificus* cases were reported in 2008, and the largest number in 2017. In general, cases increased on an annual basis. Specifically, in 2008, n = 15 cases were reported, and represent a starting point of 100 percent of the cases reported in Florida as a baseline. In 2017, n = 50 cases were reported, for an increase of 35 cases in those nine years. This resulted in an overall 233% rise in case reporting between the lowest (2008) and highest (2017) reporting yearly incidence in Florida.
(DOCX)
