# Supplementary material for: Meteorological associations of Vibrio vulnificus clinical infections in tropical settings: Correlations with air pressure, wind speed, and temperature
Source: PLoS Negl Trop Dis. 2023 Jul 6;17(7):e0011461. doi: 10.1371/journal.pntd.0011461 (PMC10353818; doi:10.1371/journal.pntd.0011461)
Supplement: S1 Fig — Here we demonstrate that the fewest V. vulnificus cases were reported in 2008, and the largest number in 2017. In general, cases increased on an annual basis. Specifically, in 2008, n = 15 cases were reported, and represent a starting point of 100 percent of the cases reported in Florida as a baseline. In 2017, n = 50 cases were reported, for an increase of 35 cases in those nine years. This resulted in an overall 233% rise in case reporting between the lowest (2008) and highest (2017) reporting yearly incidence in Florida. (DOCX) [file pntd.0011461.s001.docx]

**SUPPPORTING INFORMATION**Meteorological associations of *Vibrio vulnificus* clinical infections in tropical settings: Correlations with air pressure, wind speed, and temperature

Ayala et al. 2023


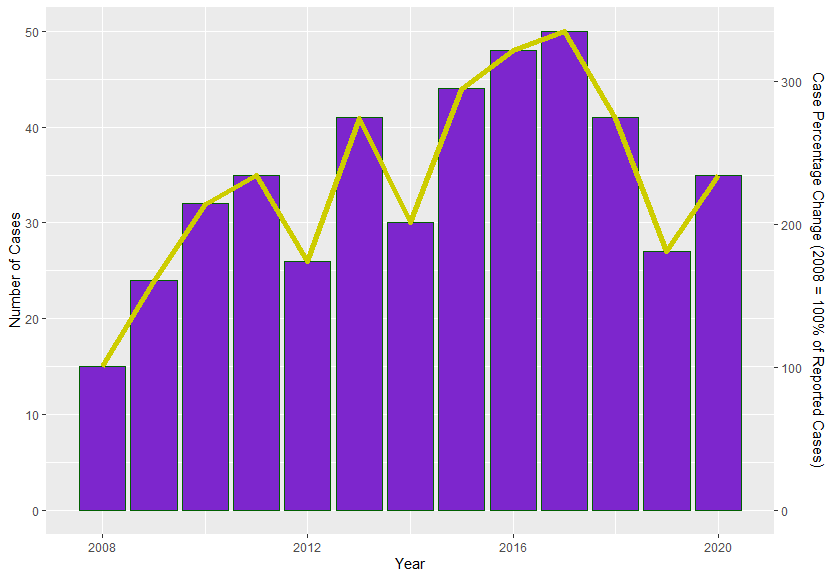


**Fig. S1.** **Vibrio vulnificus cases and percentage change over time.** Here we demonstrate that the fewest Vibrio vulnificus cases were reported in 2008, and the largest number in 2017. In general, cases increased on an annual basis. Specifically, in 2008, n = 15 cases were reported, and represent a starting point of 100 percent of the cases reported in Florida as a baseline. In 2017, n = 50 cases were reported, for an increase of 35 cases in those nine years. This resulted in an overall 233% rise in case reporting between the lowest (2008) and highest (2017) reporting yearly incidence in Florida.
